# Retraction: Tan, G., et al. Dinitrosopiperazine-Mediated Phosphorylated-Proteins Are Involved in Nasopharyngeal Carcinoma Metastasis. *Int. J. Mol. Sci.* 2014, *15*, 20054–20071

**DOI:** 10.3390/ijms21239014

**Published:** 2020-11-27

**Authors:** International Journal of Molecular Sciences Editorial Office

**Affiliations:** MDPI, St. Alban-Anlage 66, 4052 Basel, Switzerland; ijms@mdpi.com

The journal retracts the article, Dinitrosopiperazine-Mediated Phosphorylated-Proteins Are Involved in Nasopharyngeal Carcinoma Metastasis [1] cited above. 

Following publication, concerns were brought to the attention of the publisher regarding the figures. Some stained tissue pictures in Figure 6 [1] are duplicates, appearing in Figure 4A of another published paper [2]. The figure duplication brings uncertainty regarding the scientific conclusions. Therefore this paper [1] is retracted and shall be marked accordingly. 

We apologize to our readership that this went undetected until now.
